# Role of Rice Stripe Virus NSvc4 in Cell-to-Cell Movement and Symptom Development in *Nicotiana benthamiana*

**DOI:** 10.3389/fpls.2012.00269

**Published:** 2012-12-07

**Authors:** Yi Xu, Xueping Zhou

**Affiliations:** ^1^State Key Laboratory of Rice Biology, Institute of Biotechnology, Zhejiang UniversityHangzhou, China

**Keywords:** rice stripe virus, movement, chloroplast, tubules

## Abstract

Our previous work has demonstrated that the NSvc4 protein of *Rice stripe virus* (RSV) functions as a cell-to-cell movement protein. However, the mechanisms whereby RSV traffics through plasmodesmata (PD) are unknown. Here we provide evidence that the NSvc4 moves on the actin filament and endoplasmic reticulum network, but not microtubules, to reach cell wall PD. Disruption of cytoskeleton using different inhibitors altered NSvc4 localization to PD, thus impeding RSV infection of *Nicotiana benthamiana*. Sequence analyses and deletion mutagenesis experiment revealed that the N-terminal 125 amino acids (AAs) of the NSvc4 determine PD targeting and that a transmembrane domain spanning AAs 106–125 is critical for PD localization. We also found that the NSvc4 protein can localize to chloroplasts in infected cells. Analyses using deletion mutants revealed that the N-terminal 73 AAs are essential for chloroplast localization. Furthermore, expression of NSvc4 from a Potato virus X (PVX) vector resulted in more severe disease symptoms than PVX alone in systemically infected *N. benthamiana* leaves. Expression of NSvc4 in *Spodoptera frugiperda* 9 cells did not elicit tubule formation, but instead resulted in punctate foci at the plasma membrane. These findings shed new light on our understanding of the movement mechanisms whereby RSV infects host plants.

## Introduction

Rice stripe disease is the most devastating viral disease of rice in China, Japan, and Korea (Wei et al., 2009). The causal agent, *Rice stripe virus* (RSV), is the type member of the *Tenuivirus* genus and the viral genome consists of four single-stranded RNA segments (RNAs 1, 2, 3, and 4; Hibino, 1996). RNA 1 is negative-sense and encodes a putative RNA-dependent RNA polymerase. RNAs 2, 3, and 4 are ambisense, and each of which encodes two open reading frames (ORFs) with one on viral RNA (vRNA) and another on viral complementary RNA (vcRNA). RSV vRNA 2 encodes a membrane-associated protein that reportedly is an RNA silencing suppressor and interacts with SGS3 (Du et al., 2011). The vcRNA 2 encodes a glycoprotein with unidentified functions (Zhao et al., 2012). The vRNA 3 and vcRNA 3 encode a gene silencing suppressor and a nucleocapsid (NC) protein, respectively (Hibino, 1996; Xiong et al., 2009). RSV vRNA 4 encodes a disease-specific protein that accumulates in both infected plant and insect cells (Toriyama, 1986). The protein encoded by vcRNA 4 was identified as the RSV movement protein (MP; Xiong et al., 2008). RSV is transovarially transmitted by small brown planthopper (SBPH), *Laodelphax striatellus*, in a circulative-propagative manner (Falk and Tsai, 1998; Li et al., 2011). After RSV infection, rice plants often show chlorotic stripes in the newly expanded leaves, and the stripes progress into pale streaks in infected plant leaves. Because of global environment changes and the extensive increases in distribution of the transmission vector (*L. striatellus*) in the south and southeastern parts of China, RSV has caused significant losses in rice production in the past decade.

To infect a host plant successfully, viruses must overcome two obstacles; they must be capable of replicating in host cells and moving between cells and then be able to move systemically throughout the plant via the vasculature. To carry out these functions, viruses encode MPs that often interact with viral genomic (g) RNAs to form ribonucleoprotein complexes that mediate intra- and inter-cellular movement. At the plasmodesmata (PD), the MPs modify PD size exclusion limits to enable transit of the ribonucleoprotein complexes to adjacent cells. In some examples, the viral MPs form tubules that penetrate through the PD and serve as conduits for whole virus cell-to-cell transport. It has been shown that viruses often co-opt plant cellular processes to carry out specific functions required for infection (Scholthof, 2005; Shen et al., 2011). In addition to host factors that interact directly with viral MPs (Paape et al., 2006; Shimizu et al., 2009), host cytoskeleton, and endoplasmic reticulum (ER) networks also play critical roles in virus movement in hosts (Ashby et al., 2006; Harries et al., 2010). For example, both microtubules and microfilaments have been implicated in supporting cell-to-cell movement of *Tobacco mosaic virus* (TMV) in *Nicotiana benthamiana* (Brandner et al., 2008; Harries et al., 2009b, 2010). The MP of *Abutilon mosaic virus* (AbMV) is known to have an anchor domain that allows the MP to localize to the ER (Aberl et al., 2002). Association of viral MPs with the secretory pathway was also reported for viruses whose MPs form tubules. For example in cells infected with *Cowpea mosaic virus* or *Cauliflower mosaic virus*, tubule formation was independent of microtubules or microfilaments, but tubule formation required a functional secretory pathway (Huang et al., 2000; Pouwels et al., 2002). Trafficking of P3N-PIPO and CI of *Turnip mosaic virus* (TuMV) to PD has also been shown to be dependent on the host secretory pathway (Wei et al., 2010b). Interestingly, in *Grapevine fanleaf virus* infected cells both the secretory pathway and the cytoskeleton networks were reported to be involved in tubule formation and in intra-cellular targeting of virions (Laporte et al., 2003). Thus, plant virus may utilize the host cytoskeleton, the ER network, or both for PD targeting. Genomes of plant viruses are small and each virus encodes only a few proteins. Consequently, virus-encoded proteins are often multi–functional proteins. For example, the coat protein of *Turnip crinkle virus* (TCV) not only functions in movement between cells and in virion assembly, but also functions as a suppressor of gene silencing (Qu et al., 2003; Cao et al., 2010). Viral MPs also have varied functions: BC1 of AbMV accumulates preferentially at the cell periphery or around the nucleus in plant cells, and hence may participate in distinct functions (Zhang et al., 2001, 2002). The *Barley stripe mosaic virus*-encoded triple-gene block (TGB) 1 protein has similar localization patterns, and TGB2 can localize to both ER membranes and chloroplasts, indicating it also has distinct functions (Torrance et al., 2006; Lim et al., 2009). Several other viral MPs have been reported to accumulate in chloroplasts and are considered to have important roles in virus replication, viral transport, or symptom development. For example, mutation of the chloroplast-targeting signal in the *Alternanthera mosaic virus* (AltMV) TGB3 impaired the virus cell-to-cell movement and eliminated the long distance movement of the virus (Lim et al., 2010). A number of biochemical and subcellular localization activities are associated with the TGB proteins of other flexiviruses, including intra-cellular targeting, gene silencing activities, and host membrane remodeling (Verchot-Lubicz et al., 2010; Tilsner et al., 2012). The 66 K protein of Turnip yellow mosaic virus (TYMV) was reported to localize to virus-induced chloroplastic membrane vesicles, which are thought to function as TYMV RNA replication factories (Prod’homme et al., 2003). The TuMV 6 K also has been shown to target chloroplasts to result in aggregation and elicitation of membrane invaginations (Wei et al., 2010a). Former work demonstrated that NSvc4 rely on the early secretory pathway and actin-myosin VIII motility system for plasmodesmatal localization and could induce foliar necrosis from a TMV-NSvc4 hybrid vector (Yuan et al., 2011; Zhang et al., 2012). Here we present new evidence indicating that NSvc4 exerts its movement functions by trafficking on actin filaments and ER networks to reach the PD and shown that the N-terminal 125 amino acids (AAs) determine the PD localization. We also demonstrate that the NSvc4 protein targets chloroplasts in infected cells and is a symptom determinant in plant.

## Materials and Methods

### Plasmids constructions

The full length ORFs of NSvc4 protein and the N- and C-terminal deletion mutants were amplified from pBin438-NSvc4 (Xiong et al., 2008) using the Phusion High-Fidelity DNA polymerase (New England Biolabs, Ipswich, USA). The NSvc4 deletion mutant (lacking AAs 106–125) was first amplified via an overlap PCR method with the primers MP-Fol and MP-Rol (See Table A1 in Appendix for all the primers used in this study). The resulting PCR fragments were ligated individually into the pCHF3-eGFP plasmid and used for agroinfiltration into *N. benthamiana* (Xiong et al., 2008). To construct Potato virus X (PVX) NSvc4 expression vectors, full length and deletion mutants of NSvc4 were PCR amplified with primers containing *Cla*I and *Sal*I restriction sites. The PCR fragments were cloned individually into the pGEM-Teasy vector. After digestion using the *Cla*I and *Sal*I enzymes, the resulting fragments were ligated individually to the PVX pgR107 vector (provided by Dr D. C. Baulcombe, Sainsbury Laboratory, John Innes Centre, Norwich Research Park, Norwich, UK). All the plasmids were verified by DNA sequencing before further use.

### Plant inoculation and confocal microscopy

*N. benthamiana* plants were grown in a growth chamber set at 25 ± 1°C and 16 h light and 8 h dark conditions. RSV infectivity trials were carried out by rub-inoculating leaves with crude extracts from RSV-infected *O. sativa* leaves ground in phosphate buffer (0.2 M). After a 12-h incubation in the dark, the plant were transferred to a culture room set at 25 ± 1°C, 80% relative humidity, and 16 h light and 8 h dark cycle. Local and systemic leaf infections were evaluated at 3, 7, and 10 days post inoculation by RT-RCR (data not shown). Leaves of 4-week-old plants were infiltrated with *Agrobacterium tumefaciens* (strain GV3101) harboring either the full length NSvc4 sequence or one of the mutant NSvc4 plasmids using needleless syringes as described previously (Batoko et al., 2000). Leaf tissue was harvested at 48 h post agro-infiltration and examined for GFP fluorescence under a Leica TCS SP5 confocal microscope equipped with a 20× objective lens. Conditions set to excite GFP and monitor the emission were as described by Brandizzi et al. (2002). Chloroplast autofluorescence was detected using a 670-nm emission filter according to the manufacturer’s instructions. Confocal images were processed using the LCS Lite Leica software.

### Inhibitor treatments

Latrunculin B (LatB), oryzalin, and brefeldin A (BFA) were purchased from Sigma-Aldrich (St. Louis, USA) and dissolved in dimethyl sulfoxide (DMSO) to make stock solutions at 10 mM, 2 mM, and 200 μg/ml, respectively. Immediately prior to use, the stocks were diluted to 5 μM LatB, 50 μM oryzalin, and 50 μg/ml BFA using double-distilled water (ddH_2_O). Three hours before agroinfiltration, diluted LatB, oryzalin, or BFA solutions were infiltrated into *N. benthamiana* leaves using needleless syringes as described (Harries et al., 2009a). Diluted DMSO (1:1000 in ddH_2_O) was infiltrated into *N. benthamiana* leaves and used as a control. The MAN1-RFP (from soybean, which is known to localize to cis-Golgi) was used to monitoring BFA in function in our system (data not shown).

For virus inoculation assays, leaves of six-to-eight leaf stage *N. benthamiana* were rub-inoculated with 5 μM LatB, 50 μM oryzalin, or diluted DMSO (1:1000 in ddH_2_O). One day after the chemical treatments, the leaves were rub-inoculated as described previously with crude extracts prepared from RSV-infected *O. sativa* leaves (Xiong et al., 2008). After 12 h incubation in the dark, the plants were transferred to a culture room set at 25 ± 1°C, 80% relative humidity, and 16 h light (5000 lux) and 8 h dark.

### Immunocytochemistry and electron microscopy

Small tissues (approximately 1 mm wide and 3 mm long) were excised from *N. benthamiana* leaves agroinfiltrated with the bacteria harboring the pgR107 or pgR107-NSvc4 vectors. Harvested tissues were fixed with 50 mM phosphate-buffered saline (PBS), pH 6.8, containing 1% glutaraldehyde and 2% formaldehyde for 3 h at 4°C. After dehydration in a graded series of ethanol (30, 50, 70, 90, and 100%), the fixed samples were embedded in Lowicryl K4M resin as described previously (Xiong et al., 2008).

### Construction of baculovirus plasmids and transfection of Sf-9 cells

The full length *NSvc4* sequence was PCR amplified from the pgR107-NSvc4 using the primers MP-(BamH1)-F and MP-(Sal1)-R. The amplified fragments were digested with the *Bam*HI and *Sal*I restriction enzymes, and then inserted between the *Bam*HI and *Sal*I sites within the pFastBacHTGFPT transfection vector (kindly supplied by Chuanxi Zhang, Zhejiang University, Zhejiang, China) under the control of the polh promoter. The recombinant plasmid pFastBacHTGFPT-NSvc4 was transformed into *E. coli* *DH10Bac* as instructed (Invitrogen, Carlsbad, USA). After transformation, the gene cassette from the recombinant plasmid was transferred to the bacmid genome by site-specific transposition and the recombinant bacmid DNA was then isolated following the manufacturer’s instructions.

Recombinant bacmid DNA was transfected into 1.0 × 10^6^ cells *Spodoptera frugiperda* 9 (Sf-9) cell using Cellfectin Reagent (Invitrogen, Carlsbad, USA), and transfected cells were incubated at 27°C for 72 h. Supernatant of culture medium TNM-FH (Sigma-Aldrich, USA) was collected from the transfected Sf-9 cell cultures and transferred to fresh Sf-9 cells followed by an additional 48–72 h incubation period before confocal microscopy observations.

### Computational analyses of the NSvc4 protein

The transmembrane helices of NSVc4 were predicted using the Membrane Protein Explorer (MPEx, version 3.2^1^) and the ΔG prediction server^2^ (Hessa et al., 2007; Snider et al., 2009). The ΔG server provides predictions of the corresponding apparent free energy differences. In principle, a negative ΔG value by the Sec translocon predicts that a protein sequence has a TM helix and that proteins can integrate into membranes.

## Results

### Actin filaments and Golgi apparatus disruption abolish PD localization of NSvc4 and delay RSV systemic infections in *N. benthamiana*

**Figure 1:**
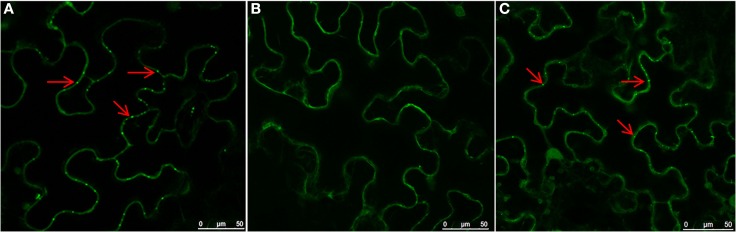
**Role of actin filaments in PD localization of NSvc4**. *N. benthamiana* leaves were first infiltrated with DMSO **(A)**, LatB **(B)**, or oryzalin **(C)**. Three hours later, the leaves were agroinfiltrated with bacteria harboring the NSvc4-eGFP vector. Infiltrated leaves were sampled at 48 h after agro-infiltration and subjected to examination under the confocal microscopy. Arrows indicate PD localization of fusion proteins.

**Figure 2:**
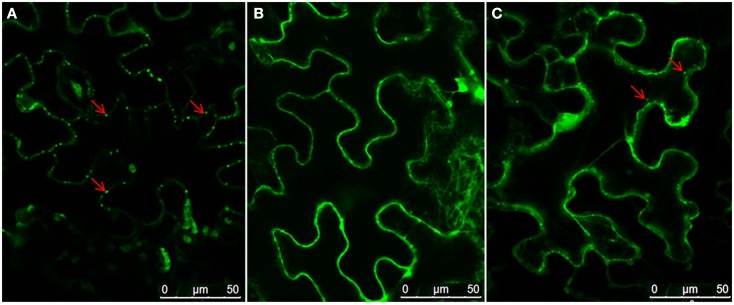
**Role of the ER-Golgi secretion pathway in PD localization of NSvc4**. *N. benthamiana* leaves were infiltrated with DMSO **(A)** or BFA **(B)**. After 3 h, the leaves were agroinfiltrated for expression of NSvc4-eGFP. The leaf shown in **(C)** was agroinfiltrated with deletion mutant of NSvc4 (NSvc4_106–125_-eGFP). Arrows indicate the localization of fusion protein.

To investigate roles of the cytoskeleton and secretory membranes in NSvc4 intra-cellular transport and PD targeting, three pharmacological inhibitors were applied to *N. benthamiana* leaves before agroinfiltration-mediated transient expression of NSvc4-eGFP. LatB and oryzalin treatments were used to disrupt actin filaments and microtubules, as previously reported (Harries et al., 2009a; Yuan et al., 2011). Expression of NSvc4-eGFP under control of the PVX pgR107-NSvc4 vector in the DMSO (control) treated *N. benthamiana* leaves yielded punctate spots at the PD (Figure 1A). When NSvc4-eGFP was expressed in LatB treated *N. benthamiana* leaves, the number of punctate spots at the PD were clearly reduced, and fluorescence was more generally dispersed throughout the cell (Figure 1B), indicating that a functional actin cytoskeleton is important for targeting NSvc4-eGFP to punctate foci at the PDs. When the NSvc4-eGFP was expressed in the oryzalin treated *N. benthamiana* leaves, abundant punctate spots similar to those noted in the DMSO controls were evident at the PD (Figure 1C). These experiments indicate that depolymerizing microtubules does not have obvious interference on formation of punctate spot at the PD. BFA is known to interfere with the ER/Golgi secretory pathway by inhibiting COPI vesicle production (Tse et al., 2006). We therefore treated *N. benthamiana* leaves with BFA and noted the NSvc4-eGFP fluorescence was more generally distributed in the cytosol than in the DMSO treated controls and that the localization at the PD and the peripheral membranes was greated reduced (Figures 2A,B). These results thus suggest that an intact Golgi secretory system has a substantial positive effect on PD targeting of NSv4-eGFP.

In order to determine whether the pharmacological affects were correlated with RSV infection, we inoculated *N. benthamiana* leaves with extracts from RSV-infected rice. We had noted earlier (Xiong et al., 2008) that RSV results in systemic infections in *N. benthamiana* after mechanical inoculation. Therefore to test the effects of the DMSO, LatB, and oryzalin treatments on RSV infection, we inoculated RSV from infected rice extracted to *N. benthamiana* leaves 1 day after application of the drug treatments. The results show that disruption of actin filaments using LatB strongly inhibits systemic infection of RSV, whereas oryzalin treatments were similar to those of DMSO on RSV systemic infection in *N. benthamiana* (Table 1).

**Table 1:** **Effect of different inhibitors on RSV infection in *N. benthamiana***.

Treatment	3 dpi^a^ inoculation leaf	3 dpi systemic leaf	7 dpi systemic leaf	10 dpi systemic leaf
LatB	15/15^b^	0/15	3/15	3/15
Oryzalin	15/15	0/15	11/15	12/15
DMSO	15/15	0/15	13/15	15/15

*^a^Days post inoculation of RSV in *N. benthamiana*. ^b^ The denominator shows the number of *N. benthamiana* plants used in these treatment; the numerator represents the number of *N. benthamiana* plants with symptom*.

### Computational analysis of NSvc4 and domains responsible for NSvc4 PD localization

**Figure 3:**
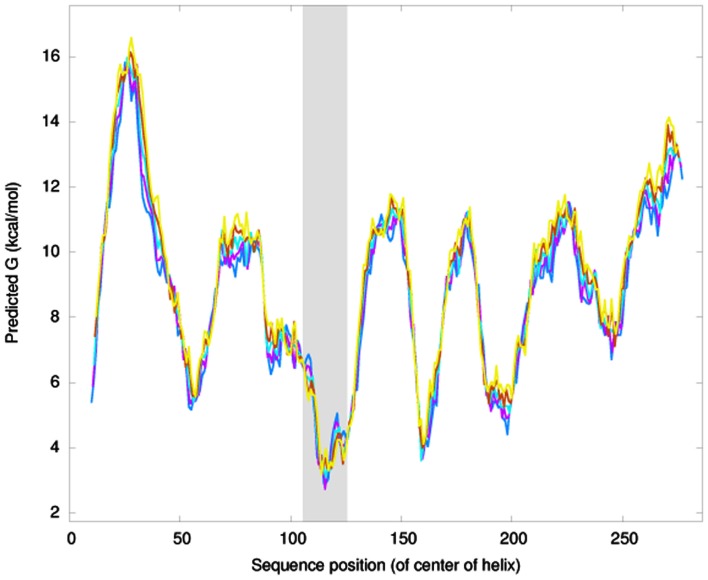
**NSVc4 transmembrane domain prediction**. Prediction of transmembrane domain was carried out with the ΔG prediction server (http://dgpred.cbr.su.se/index.php?p=home). The *Y*-axis shows the predicted G value and the *x*-axis represents the amino acid sequence position. The dark region spanning amino acids 106–125 is predicted to be a transmembrane domain.

Using the Membrane Protein Explorer program, AAs spanning positions 106–125 of the NSvc4 protein have properties of a transmembrane domain (Figure 3). To confirm this prediction, we deleted AAs 106–125 from the NSvc4 to create NSvc4_Δ106–125_-eGFP, and expressed the mutant transiently from the PVX vector in the epidermal cells of *N. benthamiana* leaves via agro-infiltration. Confocal microscopy observations revealed that PD localization by the deletion mutant was substantially reduced compared to the DMSO controls. Only a few apparently intact foci were evident and most of the fluorescence was diffuse and appeared to be associated with the cytosol (Figure 2C). These observations provide evidence suggesting that the predicted transmembrane region (AAs 106–125) in the NSvc4 protein provides an important anchor domain that is required for NSvc4 trafficking on the endomembrane network.

**Figure 4:**
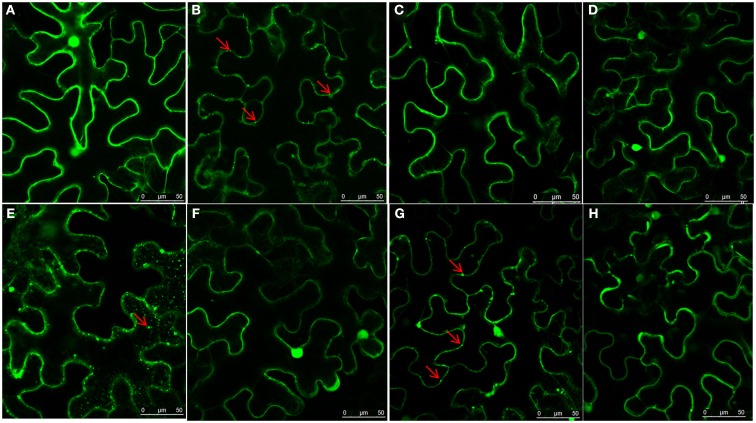
**Determination of domains within the NSvc4 that are responsible for PD localization**. Tissue was collected from *N. benthamiana* leaves at 48 h after agroinfiltration with pCHF3-eGFP **(A)**, pCHF3-NSvc4-eGFP **(B)**, pCHF3-NSvc4_1–54_-eGFP **(C)**, pCHF3-NSvc4_1–73_-eGFP **(D)**, pCHF3-NSvc4_Δ106–125_-eGFP **(E)**, pCHF3-NSvc4_1–106_-eGFP **(F)**, pCHF3-NSvc4_1–125_-eGFP **(G)**, and pCHF3-NSvc4_125–286_-eGFP **(H)**. Harvested leaf samples were examined by confocal microscopy. Arrows indicate the localization of NSvc4 and its mutant fusion protein.

To determine the domain responsible for NSvc4 PD localization, a series of NSvc4 deletion mutants were constructed, inserted into the pCHF3 vector and expressed transiently by agroinfiltration into *N. benthamiana* leaf cells. The fluorescence patterns in cells at 48 h after infiltration revealed that the NSvc4_1–54_-eGFP, NSVc4_1–73_-eGFP, NSvc4_1–106_-eGFP, and NSvc4_Δ125–286_-eGFP mutant derivatives each elicited GFP expression patterns similar to those produced by pCHF3-eGFP, the GFP control vector (Figure 4). However, fluorescence from the NSvc4_1–125_-eGFP deletion mutant protein accumulated in punctate foci at the PD that appeared to be similar to the fluorescence elicited in cells expressing NSvc4-eGFP (Compare Figures 4B,G). In marked contrast, the NSvc4_Δ 106–125_-eGFP proteins accumulated as small punctate bodies in the cytoplasm and were not observed at the PD (Figure 4E). These observations indicate that the N-terminal 1–125 AA fragment contains a PD localization signal that is sufficient for PD targeting.

### Localization of NSvc4 in sphere-like compartments and chloroplast

**Figure 5:**
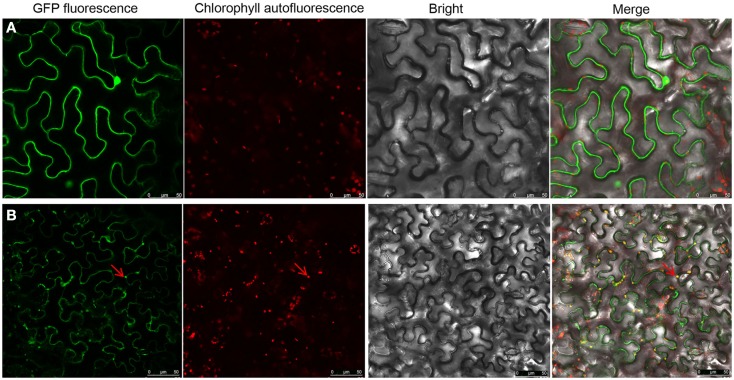
**Localization of NSvc4 proteins in chloroplast**. Tissues were harvested from *N. benthamiana* leaves at 48 h after agroinfiltration with **(A)** pCHF3-eGFP or **(B)** pCHF3-NSvc4-eGFP. The harvested tissues were then examined under a confocal microscope. Fluorescence emissions were collected simultaneously, with a one photon multiplier-tube bandwidth set at 500–530 and 660–700 nm, respectively. Arrows indicate the sphere-like compartments formed by NSvc4-eGFP fusion protein.

Image analysis indicated that as well as localizing at the PD, the NSvc4 protein also accumulated in discrete, sphere-like compartments of approximately 4 mm in diameter in cells (Figure 5). To determine the subcellular localization of these spheres, epidermal cells expressing NSvc4-eGFP were analyzed by confocal microscope. A lambda scan set at 5 nm intervals between 595 and 755 nm for analysis of the sphere-like compartments had emission peaks at 500–530 nm and at 650–700 nm (Figure 5B). The spectral characteristics of the 650–700 nm emission peak were similar to the chlorophyll spectrum (maximum at 680 nm). So, the fluorescence spectra were collected simultaneously, with one photon multiplier-tube bandwidth set at 500–530 nm and a second one at 660–700 nm, and the NSvc4-eGFP and chlorophyll autofluorescence was merged to generate yellow fluorescent of the overlapping foci (Figure 5).

**Figure 6:**
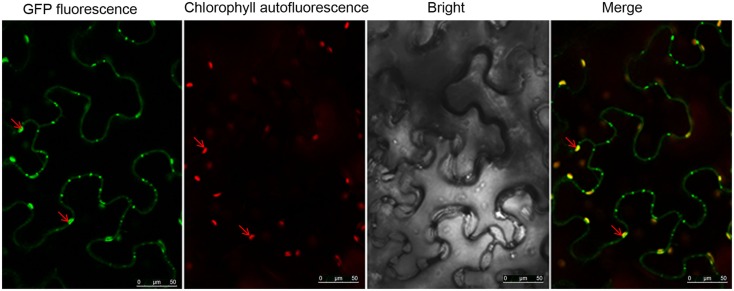
**NSvc4-eGFP expressed using the Potato virus X-based vector localized to chloroplasts**. At 7 or 8 days post inoculation with pgR-NSvc4-eGFP, systemically infected leaves were sampled and examined by confocal microscope. Arrows indicate the sphere-like compartments formed by NSvc4-eGFP fusion protein.

To confirm the presence of NSvc4 in the chloroplasts, NSvc4-eGFP was expressed in *N. benthamiana* leaf cells using the PVX vector (pgR107). By 7–8 days post agroinfiltration (dpi), leaves with systemic symptoms were sampled and examined by confocal microscopy. In cells expressing the NSvc4-eGFP, the GFP signal co-localized with the chlorophyll autofluorescence, suggesting that a fraction of the expressed NSvc4-eGFP protein accumulates in the chloroplasts (Figure 6).

**Figure 7:**
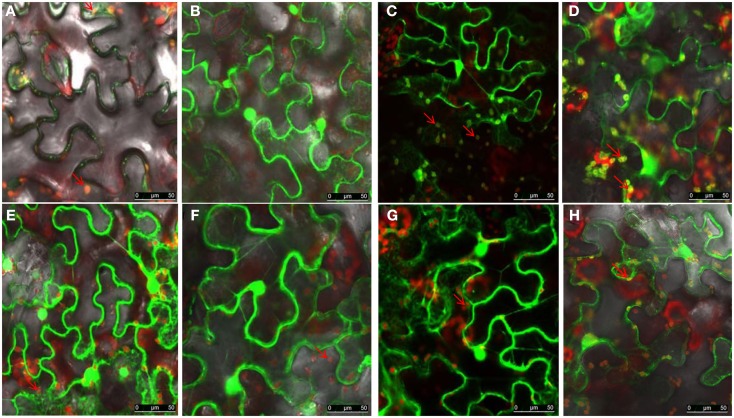
**The N-terminus 73 amino acids determine the chloroplast localization of NSvc4**. *N. benthamiana* leaves were agroinfiltrated with pCHF3-NSvc4-eGFP **(A)**, pCHF3-NSvc4_1–54_-eGFP **(B)**, pCHF3-NSvc4_1–73_-eGFP **(C)**, pCHF3-NSvc4_1–125_-eGFP **(D)**, pCHF3-NSvc4_54–73_-eGFP **(E)**, pCHF3-NSvc4_125–286_-eGFP **(F)**, pCHF3-NSvc4_106–125_-eGFP **(G)**, pCHF3-NSvc4_Δ106–125_-eGFP **(H)**. At 48 h post agroinfiltration the leaves were sampled and examined under a confocal microscope. The fluorescence emission was collected simultaneously, with one photon multiplier-tube bandwidth set at 500–530 and 660–700 nm, respectively. Arrows indicate the sphere-like compartments and the chloroplast.

To determine which region of NSvc4 is required for chloroplast-targeting, we agroinfiltrated plasmids harboring the wild type or mutant NSvc4-eGFP fusions (Figure 7). The results showed that NSvc4_1–73_-eGFP accumulated in the sphere-like compartments and in the chloroplasts of the epidermal leaf cells (Figure 7B). However, NSvc4_1–54_-eGFP and NSvc4_54–73_-eGFP localized around the nuclei and in the cytoplasm, but were not obvious in the chloroplasts (Figures 7A,E). The remaining NSvc4 mutants localized exclusively in the cytoplasm. These observations suggest that the N-terminal 73 AAs contain a chloroplast-targeting signal.

### The NSvc4 PVX vector induces more severe symptoms in *N. benthamiana* than PVX

**Figure 8:**
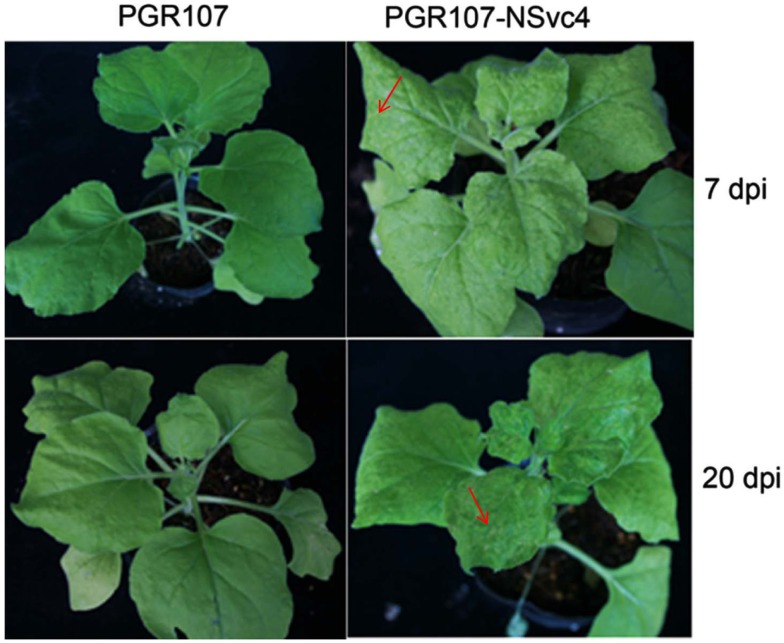
**Symptom expression in *N. benthamiana* plants after infection with the wild type PVX vector or the NSVc4 expressing PVX vector**. *N. benthamiana* plants were infiltrated with pgR107 (wild type PVX) or pgR107-NSvc4 (PVX expressing NSvc4) at 7 or at 20 dpi. Allows showed the foliar necrosis in pgR107-NSvc4 infected *N. benthamiana*.

**Figure 9:**
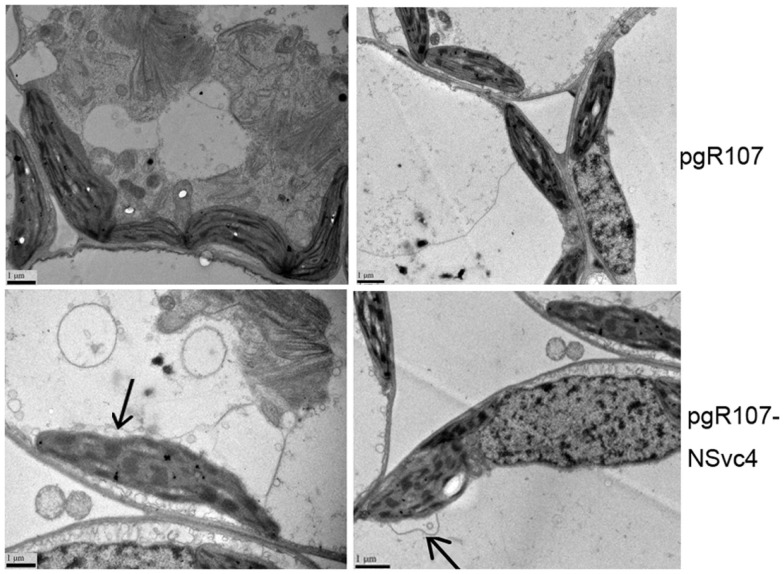
**Ultrastructural changes resulting from infection with wild type PVX or PVX-NSvc4**. Tissue sections were prepared from the leaves infected with the wild type PVX or PVX expressing NSvc4. Morphological changes in chloroplast were only observed in plants infected with PVX expressing NSvc4. Arrows indicate alterations of chloroplast membrane.

**Figure A1:**
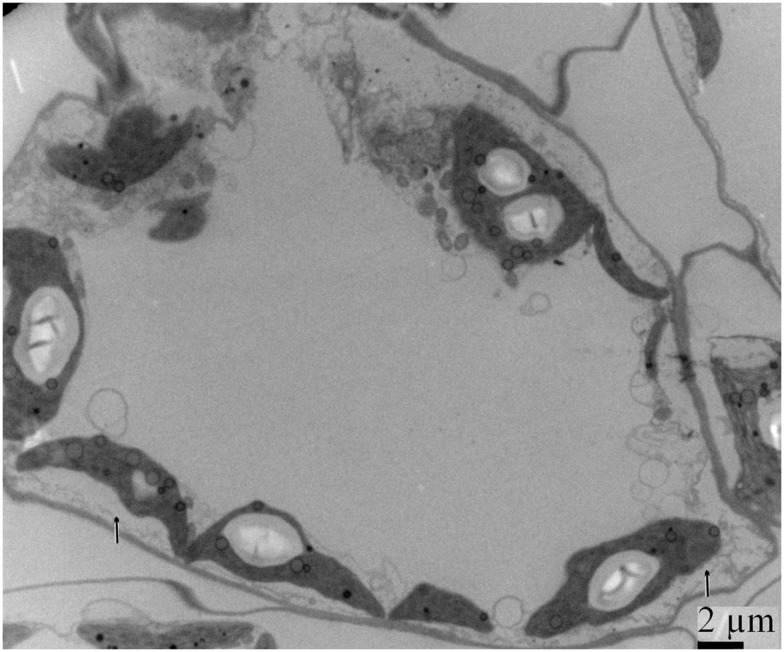
**Ultrastructural structures resulting from 20 days post rub-inoculating *N. benthamiana* leaves with crude extracts from RSV-infected *O. sativa* leaves**. Allows showed that membrane proliferations radiating from the chloroplasts into the cytoplasm.

By 7 days after agroinfiltration of *N. benthamiana* plants for expression of the wtPVX vector (pgR107) or the NSvc4 (pgR-NSvc4), all plants developed systemic symptoms in the upper emerging leaves. Symptoms in plants infiltrated with pgR-NSvc4 were more severe than in plants infiltrated with the wtPVX vector. By 20 dpi, virus symptoms in plants infected with wtPVX vector disappeared, whereas symptoms in the PVX-NSvc4 infected plants remained intense and developed foliar necrosis (Figure 8). Reverse transcription PCR result showed that NSvc4 was accumulated in leaves of the PVX-NSvc4 infected plants in both the early and the late infection stages (data not shown). Examination of thin sections prepared from the PVX or PVX-NSvc4 infected *N. benthamiana* leaf tissues by electron microscopy revealed major malformations of chloroplast grana and electron lucent bodies beneath the membranes of PVX-NSvc4 infected cells, but similar malformations were not evident in wtPVX infected cells. In addition, proliferations radiating from the chloroplasts into the cytoplasm were observed in the PVX-NSvc4 and RSV rub-inoculating infected *N. benthamiana* leaves, but not in the cells infected with wtPVX (Figure 9 and Figure A1 in Appendix).

### Symptom development is independent of NSvc4 chloroplast localization

**Figure 10:**
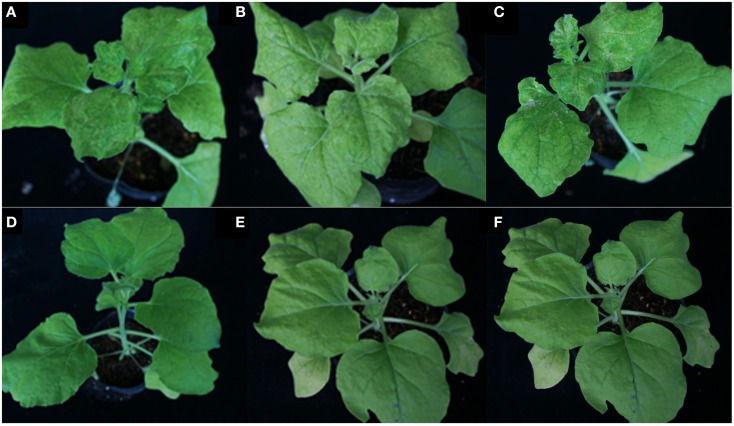
**Symptoms elicited by wild type PVX or PVX expressing mutants of NSvc4**. *N. benthamiana* plants were agroinfiltrated with various PVX constructs. Plant infected with PVX-NSvc4 **(A)**, PVX-NSvc4_1–125_ **(B)**, PVX-NSvc4_125–286_ **(C)**, PVX **(D)**, PVX-NSvc4_1–53_ **(E)**, and PVX-NSvc4_1–73_ **(F)**. Photographs were taken at 20 days post agroinfiltration.

To determine the correlation between NSvc4 chloroplast localization and symptom development, PVX vectors expressing various mutants of NSvc4 were agroinfiltrated individually into *N. benthamiana* leaves. The results demonstrate that NSvc4_1–73_ and NSvc4_1–106_ are capable of targeting chloroplasts (Figure 10; Table 2). Interestingly, plants infected with two mutant viruses developed phenotypes similar to those caused by the wild wtPVX at 7dpi, and the disease phenotype also recovered by 20 dpi. Interestingly, NSvc4_106–286_ was predicted not to localize to chloroplasts, but, the mutant still elicited a severe symptom phenotype in infiltrated *N. benthamiana* plants that was maintained for up to 20 dpi. These results indicate that NSvc4 chloroplast localization is dispensable for the exacerbated symptoms. Hence, it is possible that the NSvc4 transmembrane domain has a role in chloroplast malformations, membrane proliferations from the chloroplasts and symptom development.

**Table 2:** **Targeting of NSvc4 and its mutants to plasmodesmata and chloroplast and their roles in symptom development**.

Localization/symptom	Plasmodesmata	Chloroplast	Symptom
NSvc4	+	+	+
NSvc4_1–54_	–	–	–
NSvc4_1–73_	–	+	–
NSvc4_1–106_	–	+	n
NSvc4_1–125_	+	+	+
NSvc4_125–286_	–	–	+
NSvc4_106–286_	–	–	+

*n, The symptom data was not record*.

### NSvc4 protein did not mediate tubule formation in Sf-9 cells

**Figure 11:**
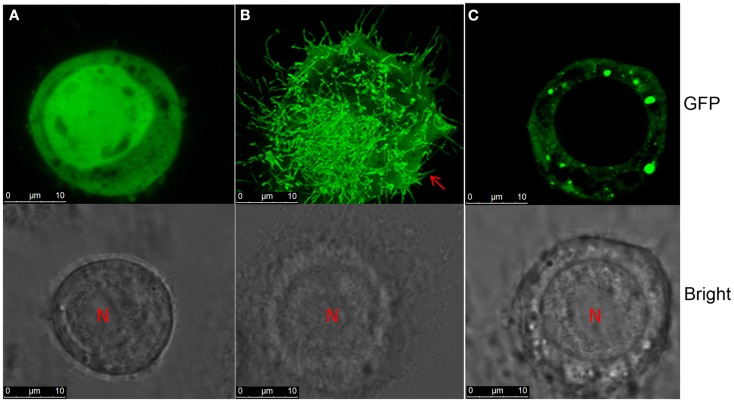
**Tubule formation in Sf-9 cells**. *S. frugiperda* 9 cells were transinfected with recombinant baculovirus AcNPV/GFP **(A)**, AcNPV/NSm-GFP **(B)**, or AcNPV/NSvc4-GFP **(C)**. Images were taken at 72 h post transfection.

Our earlier research has shown that NSvc4 accumulated at PD in the walls of RSV-infected cells (Xiong et al., 2008). Because the NSm MP of *Tomato spotted wilt virus* (TSWV) formed tubule-like structures in insect cells (Storms et al., 1995), we decided to investigate the possibility of tubule formation by RSV NSvc4. In these experiments, the TSWV NSm (AcNPV/NSm-GFP) protein elicited numerous tubule-like extensions on Sf-9 cell surface by 36–48 h post transfection. However, Sf-9 cells transfected with the RSV NSvc4 protein (AcNPV/NSvc4-GFP), failed to develop similar tubules by 48 hpi. In contrast to the free GFP protein (AcNPV/GFP), which was distributed uniformly in the nuclei and in the cytoplasm, the NSvc4-GFP protein (AcNPV/NSvc4-GFP) accumulated as globular structures at the cell periphery and, in this regard, was similar to the localization patterns of NSvc4 in plant cells (Figure 11). However, it has been reported in an abstract that the NS2 protein encoded by RSV vRNA 2 can induce tubule-like structures in insect cells (ITMGCM, 1999), but this report has not been verified in a peer reviewed paper. Nevertheless, it is possible that NS2 may interact with NSvc4 to facilitate RSV movement, so in future experiments, we plan to investigate possible roles of NS2 and NSvc4 protein interactions to determine whether they may act together to facilitate RSV cell-to-cell transport.

## Discussion

Our previous studies of RSV NSvc4 have indicated that NSvc4 belongs to the 30 K MP superfamily, and have shown experimentally that the protein interacts with single-stranded RNA *in vitro*, traffics to the PD of dicot cells and can move to adjacent cells after bombardment (Xiong et al., 2008). Within the 30 K superfamily, the TMV MP has been studied most intensively and is known to target PD via trafficking on cortical ER and actin cytoskeleton (Wright et al., 2007; Hofmann et al., 2009). A recently report indicates that targeting of NSvc4 to PDs utilizes the actin microfilament pathway and the myosin VIII rather than myosin XI motility system (Yuan et al., 2011). Our results complement and verify this study by demonstrating that actin microfilament dissociation by LatB and Golgi disruption by BFA interfere with PD targeting of the NSvc4-eGFP fusion protein, whereas disruption of microtubules by oryzalin has little effect on PD targeting. Moreover, microfilament disruption but not microtubule disruption inhibits infection of RSV in *N. benthamiana*, Thus, our combined results clearly suggest that targeting of NSvc4 to PDs depends on a functional ER and actin network.

Many viral MPs within the 30 K superfamily have a hydrophilic region at their C-termini. Deletion or alanine-scanning mutations within the C-termini of several viral MPs have demonstrated that this region is dispensable for cell-to-cell movement (Schoelz et al., 2011). We have now extended previous RSV studies through transient expression of wtNSvc4 and NSvc4 mutants in cells, and have determined that NSvc4 differs from the general MP rule because the N-terminal 125 AAs are sufficient to target the truncated fragment to the PD. Furthermore, we have shown that AAs 106–125 contain a predicted transmembrane domain and that deletion of this domain abolishes the PD targeting ability of NSvc4. These results strongly suggest that the 20 deleted residues serve as an integral membrane signal that facilitates insertion into the ER.

Several previous reports have shown that some viral MPs accumulated in chloroplast and thus might have an important role in virus replication, movement, and/or symptom development (Prod’homme et al., 2003; Torrance et al., 2006). For example AltMV TGB3 was shown to be responsible for AltMV movement between cells and contained a novel signal which was required for chloroplast membrane localization. Here we provide definitive evidence that RSV NSvc4 has a chloroplast-targeting signal within its N-terminal 73 residues, and that this signal targets the NSvc4-GFP chloroplast in both the agro-mediated and PVX-based expression systems. We anticipate further studies to elucidate the potential involvement of chloroplast-targeting in the RSV life cycle.

Expression of the NSvc4 through PVX-based vector exacerbated disease symptoms in *N. benthamiana* than the symptoms elicited by PVX alone. Electron microscope observations suggested that disease symptoms correlated with chloroplast malformations and cytoplasmic membrane proliferations in cells. However, expression of mutants of NSvc4 indicated no direct connection between chloroplast localization of NSvc4 and symptom development. We propose that the chloroplast-targeting phenomenon may be involved in RSV replication or other unidentified activities. Considering the chloroplast malformations and membrane proliferations in the PVX-NSvc4 infected *N. benthamiana* cells, it is reasonable to propose that the transmembrane activity of the NSvc4 may play a pivotal role in development of disease symptoms. Because viral MPs modify PD structures and increase PD size exclusion limits, transgenic plants expressing viral MPs often show alterations in plant development. Plant developmental anomalies have also been demonstrated through infection of *N. benthamiana* plants using TMV-based vector expression NSvc4 (Zhang et al., 2012), so the phenomena we have observed are not virus specific. The authors also found that region D17–K33 was recognized as a crucial domain for leaf necrosis response using TMV-based vector expression NSvc4 (Zhang et al., 2012). In our experiment, we also observed foliar necrosis expressed of PVX-NSvc4. More detailed work is needed to determine the regions responsible for formation of foliar necrosis expressed from PVX vector. From these accumulated data, we conclude that RSV NSvc4 is a symptom determinant that affects the host phenotype, but the mechanisms whereby the protein functions in symptom development remain to be elucidated.

One of the major questions unique to RSV movement is the form in which infectious entities might move from initial infection foci to adjacent cells. Because RSV is a negative strand “ambisense” virus, it is obvious that the NC must be involved in intra-cellular transit in order to facilitate nascent transcription and replication in newly invaded plant cells. Similar events also must function during RSV infections of planthopper vectors. Therefore, we carried out preliminary experiments to determine the location of NSvc4 and the TSWV NSm MP in insect cells. Our results show that in contrast to NSm, NSvc4 failed to produce tubule-like structures after plasmid transfection into Sf-9 cells, but instead formed large foci at the surface of the cells. We previously were unable to detect NSvc4 binding to the RSV NC protein, but have shown that NSvc4 exhibits non-specific RNA binding in gel shift assays. These results suggest that NSvc4 may be able to access RNA encapsidated in the NC, and such a mechanism is compatible with recent experimental data for Vesicular stomatitis virus (VSV), the most intensively studied negative strand virus (Green et al., 2011). Interestingly, the VSV NC (N) protein is thought to undergo conformational changes to permit access by the polymerase protein during transcription and replication. Moreover, the matrix protein of negative strand viruses has mechanisms to discriminate genomic NCs from antigenomic NCs during morphogenesis, and these likely are RNA sequence specific. Therefore, we posit that NSvc4 specifically recognizes RNA in RSV gNCs and ferries these complexes to the cell wall and then enlarges the PD complexes sufficiently to facilitate NC transit to adjacent cells. Although, different mechanisms, possibly cell fusion, may be involved in systemic spread in infected planthoppers, we envision that NSvc4:NC associations with NCs likely function during insect infection processes. Therefore, we are planning further investigations to elucidate the complicated mechanisms whereby RSV moves between plant and insect cells.

## Conflict of Interest Statement

The authors declare that the research was conducted in the absence of any commercial or financial relationships that could be construed as a potential conflict of interest.

## Footnotes

^1^http://blanco.biomol.uci.edu/mpex/

^2^http://dgpred.cbr.su.se/index.php?p=home

## Appendix

**Table A1:** **Primers used in our experiments**.

MP(Kpn1)-F	5′-ggggtaccATGGCTTTGTCTCGACTTTTG-3′
MP(BamH1)-R	5′-CGGGATCCcatgatgacagaaacttcag-3′
MP(BamH1)-R54	5′-GGATCCtgtggcagcttggtcaatc-3′
MP(BamH1)-R73	5′-GGATCCATCATACTTGTTCACCTTGACAT-3′
MP(BamH1)-R106	5′-CGGGATCCatgggtgagaggttgatg-3′
MP(kpn1)-F125	5′-gggtaccATGagtggaataactaccctcc-3′
MP(Kpn1)-F106	5′-gggtaccATGtatccattctttagagtggc-3′
MP(BamH1)-R125	5′-CGGGATCCagctctacccttgattcct-3′
MP-Fol	5′-cctctcacccatagtggaataactaccctc-3′
MP-Rol	5′-agttattccactatgggtgagaggttgatg-3′
eGFP(Sal1)-R	5′-GTCGACTTACTTGTACAGCTCGTCCAT-3′
MP(sal1)-R54	5′-GTCGACCTAtgtggcagcttggtcaatc-3′
MP(sal1)-R73	5′-GTCGACCTAATCATACTTGTTCACCTTGACAT-3′
MP(sal1)-R125	5′-GTCGACCTAagctctacccttgattcct-3′
MP(Cla1)-F125	5′-CCATCGATATGagtggaataactaccctcc-3′
MP(Cla1)-F	5′-ATCGATATGGCTTTGTCTCGACTTTT-3′
MP(sal1)-R	5′-GTCGACCTACATGATGACAGAAACTTC-3′
MP-(BamH1)-F	5′-GGATCCATGGCTTTGTCTCGACTTTT-3′
MP-(Sal1)-R	5′-GTCGACCTACATGATGACAGAAACTTC-3′
